# Emotion Identification Using Extremely Low Frequency Components of Speech Feature Contours

**DOI:** 10.1155/2014/757121

**Published:** 2014-05-20

**Authors:** Chang-Hong Lin, Wei-Kai Liao, Wen-Chi Hsieh, Wei-Jiun Liao, Jia-Ching Wang

**Affiliations:** Department of Computer Science and Information Engineering, National Central University, Taiwan

## Abstract

The investigations of emotional speech identification can be divided into two main parts, features and classifiers. In this paper, how to extract an effective speech feature set for the emotional speech identification is addressed. In our speech feature set, we use not only statistical analysis of frame-based acoustical features, but also the approximated speech feature contours, which are obtained by extracting extremely low frequency components to speech feature contours. Furthermore, principal component analysis (PCA) is applied to the approximated speech feature contours so that an efficient representation of approximated contours can be derived. The proposed speech feature set is fed into support vector machines (SVMs) to perform multiclass emotion identification. The experimental results demonstrate the performance of the proposed system with 82.26% identification rate.

## 1. Introduction

As technology advances, computers or machines with human emotion are no longer an unreachable dream. A system that understands human emotions can provide more personalized services and be extended to more applications. Professor Rosalind Picard stated that affective computing explores the topic about computing that relates to, arises from, or influences emotions. This concept creates a new computing system or idea of human-machine interface design which can realize, recognize, and utilize human emotions. The concept above coincides with “Technology derives from humanity.” Today, in the twenty-first century, human's demand for high-tech product is no longer merely some specific functionality. Humanity is an essential element for successful product. The design focusing on affectivity will become the mainstream.

Speech emotion identification [1–8] can be divided into two parts, features and classifiers. In the feature part, many features have been considered having a significant influence on emotion identification. For example, both of [1, 2] use pitch, energy, and Mel-frequency cepstral coefficients (MFCCs) and so forth. Other features such as voice quality [8], spectrum features [3], and nonlinear Teager energy operator features [9] are also exploited to represent the characteristics of emotions. In [4, 10], the authors use linear regression to describe graphical trend of pitch and use polynomial coefficients as features to train and identify [8]. Legendre trend uses polynomial bases that are different from linear regression to describe speech information and obtain features [1, 8]. In this paper, besides the statistical analysis of the selected acoustical features, approximated speech feature contours with (PCA) [11, 12] are also utilized in the feature extraction. In the classifier part, we use a multiclass support vector machine (SVM) [7, 12, 13] to achieve emotion identification.

The rest of this paper is organized as follows.  Section 2 introduces the overview of the system. The proposed speech feature set for emotion identification is illustrated in Section 3.  Section 4 describes the emotion classifier used in this paper. Next, Section 5 gives the experimental results. Conclusions are finally drawn in Section 6.

## 2. System Overview

**Figure 1:**
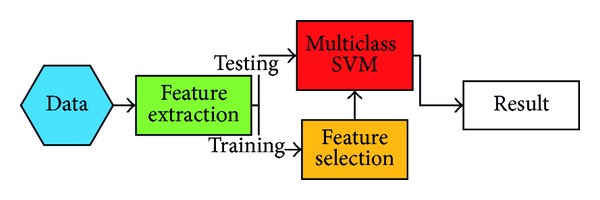
Block diagram of the proposed system.

Figure 1 illustrates the proposed system block diagram. The proposed emotion identification system can be divided into two main parts, feature extraction and emotion classifier. In the feature extraction, we extract all the acoustical features from both of training and testing speeches. The acoustical features comprise silence/active, voiced/unvoiced, pitch, log frame energy (LFE), subband power, spectral centroid, spectral bandwidth, spectral flatness, MFCCs [3], and ratio of spectral flatness to spectral centroid (RSS) [7]. The statistical analysis features are generated from the aforementioned frame-based acoustical features. The chosen feature is important for classification task [14, 15]. A feature selection procedure chooses the effective statistical analysis features to form the selected feature set. The selected statistical analysis feature set as well as the selected approximated speech feature contours is combined to form the proposed feature set. In the emotion classifier, a multiclass SVM is used as the classifier to identify the emotion class of a speech utterance.

## 3. Emotion Feature Extraction

Extracting appropriate speech features is an important issue in emotion identification. Features can be used to describe the emotion characteristics of speech. Suitable feature set can effectively increase the performance of classifiers. For emotional speech identification, most systems partition speech waveforms into segments called frames, and a classifier is trained using features extracted from frames. These features are frame-based speech features, which usually have an excellent performance for highly nonstationary signals. To further enhance the frame-based features, statistical analysis such as ratio, mean, and standard deviation of frame-based features usually creates more reliable speech features. In addition to statistical analysis of frame-based features, this paper also considers the approximated speech feature contours.

### 3.1. Frame-Based Features with Statistical Analysis

The frame-based acoustical features extracted in this paper include silence/active, voiced/unvoiced, pitch, LFE, subband power, spectral centroid, spectral bandwidth, spectral flatness, MFCCs [3], and RSS [7]. Among these frame-based acoustical features, several simpler ones are briefly explained below. Silence/active denotes if a frame is a silence frame or not, while voiced/unvoiced represents if a frame is a voiced frame. Pitch expresses the repeating duration in a voiced speech frame. LFE makes a logarithm operation on a frame energy. In the following, the more complicated frame-based acoustical features are explained.

The subband powers are extracted from specified subband intervals. In this paper, we adapt four intervals which are [0, 0.125*f*_0_], [0.125*f*_0_, 0.25*f*_0_], [0.25*f*_0_, 0.5*f*_0_], and [0.5*f*_0_, *f*_0_], where *f*_0_ is half of the sampling rate. Spectral centroid [16, 17] denotes the weighted average of the frequencies of a speech power spectrum generated from a speech frame. Spectral bandwidth [16, 17] indicates whether the shape of the speech power spectrum concentrates in the neighborhood of the spectral centroid or else spreads out over the speech power spectrum. The spectral flatness is obtained by computing the ratio of the geometric mean and the arithmetic mean of the speech power spectrum coefficients. MFCCs are nonparametric representations of audio signal, which models the human auditory perception system. The RSS proposed by Kim et al. for emotional speech recognition [7] is the ratio of spectral flatness to spectral centroid. The RSS of *i*th frame is calculated by

(1) $$\begin{array}{c} \text{RSS}\left(i\right)=\frac{1000\times \text{SF}\left(i\right)}{\text{SC}\left(i\right)}, \end{array}$$

where SF(*i*) and SC(*i*) denote spectral flatness and spectral centroid of *i*th frame.

To convert these frame-based features into more effective features, statistical analysis is applied to each of them. This paper adopts three statistical analysis types, ratio, mean, and standard deviation. The ratio analysis is applied to silence/active and voiced/unvoiced to calculate the silence ratio and voiced ratio, respectively. As for the remaining features, both mean and standard deviation analysis are performed.

### 3.2. Approximated Speech Feature Contours

**Figure 2:**
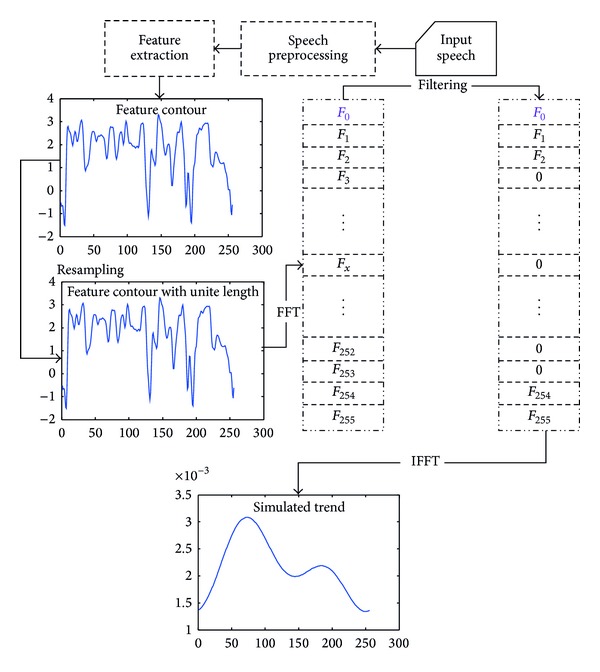
Flowchart to obtain the approximated speech feature contour.

In the previous subsection, statistical analysis of frame-based acoustical features is performed. This subsection provides another point of view to extract emotion related information, that is, the temporal shape of a feature contour. As the detailed shape information may be merely aroused from the various phone pronunciations rather than the various emotions, an approximated speech feature contour is proposed.  Figure 2 depicts the flowchart to obtain the approximated speech feature contour.

Let *x*_*lk*_ denote an original feature contour, where *l* is the speech utterance index, *k* = 1,2,…, *T*_*l*_ is the frame index, and *T*_*l*_ is the frame number of *l*th speech utterance. As the durations of different utterances differ, a resampling scheme is performed to normalize the feature contour durations so that the feature contour comparisons are fair.

Besides, speech feature contours usually contain too much local fluctuations that are unrelated to emotion expression. To lessen this impact, this paper presents the approximated speech feature contours. First, the well-known discrete Fourier transform [18] is applied to the resampled speech and transformed it to frequency domain by

(2) $$\begin{array}{c} y_{lj}=\overset{F\_\text{length}-1}{\underset{k=0}{\sum }}e^{-(2\pi i/n)jk}x_{lk}^{\prime },\;j=0,1,\ldots ,F\_\text{length}-1, \end{array}$$

where *x*′ represent the resampled feature contour and its duration has been normalized to *F*_length. Moreover, *x*_*lk*_′ refers to the *k*th feature in the feature contour of the *l*th speech utterance, and *y*_*lj*_ is the *j*th Fourier coefficient of the *l*th speech utterance.

The presented approximated speech feature contours rely on extracting extremely low frequency components to speech feature contours. To extract these frequency components, the symmetric property of discrete Fourier spectrum is also required to be taken into account. Assume *m* lowest-frequency Fourier coefficients (frequency bins) are to be extracted; the frequency component extraction is achieved by converting *y*_*lj*_ in (2) into (3):

(3) $$\begin{array}{c} y_{lj}^{\prime }=\{\begin{array}{cc} 0, & m<j<n-m,\\ y_{lj}, & \text{others}. \end{array} \end{array}$$

Finally, the extremely low frequency components *y*_*lj*_′ in (3) are transformed to time-domain by inverse Fourier transform. The resynthesized time-domain signal is the proposed approximated speech feature contour.

**Figure 3:**
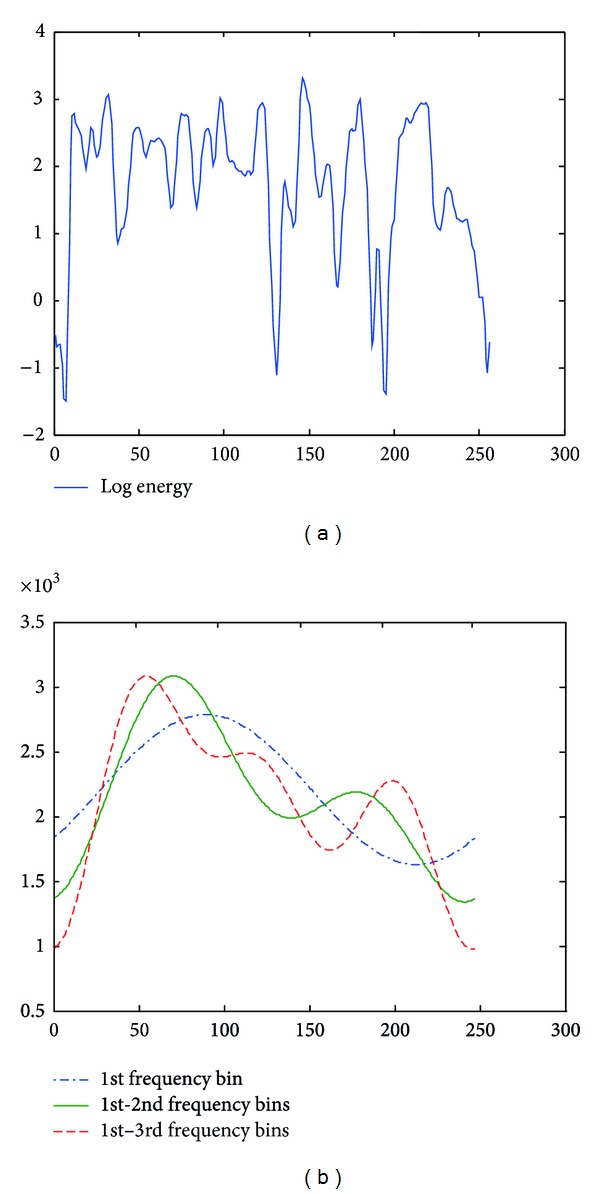
Examples of approximated LFE contours from an angry speech utterance.

**Figure 4:**
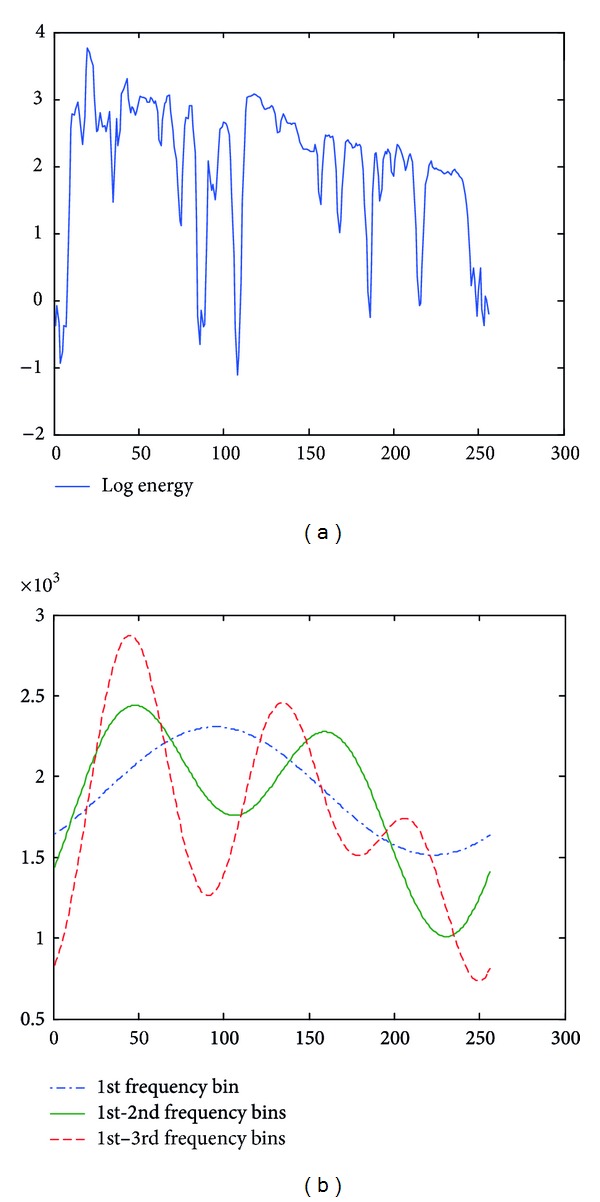
Examples of approximated LFE contours from a bored speech utterance.

**Figure 5:**
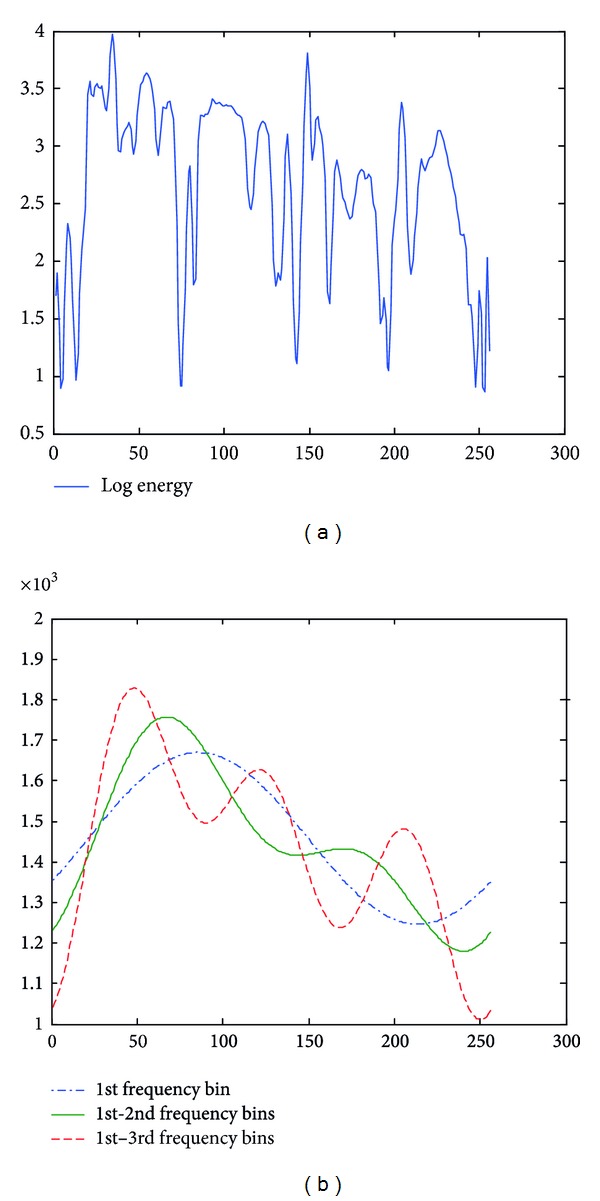
Examples of approximated LFE energy contours from a sad speech utterance.

In this paper, parameter *m* is chosen as 1, 2, and 3. Taking log energy feature, for example, Figures 3, 4, and 5 exemplify the approximated log energy contours of an angry, a bored, and a sad speech utterances, respectively.

### 3.3. Approximated Speech Feature Contours with Principal Component Analysis

In this paper, principal component analysis is adopted to represent the approximated speech feature contours in an efficient way. Principal component analysis is a well-known technique in multivariate analysis and pattern recognition [11]. In this study, PCA is used to reduce the high feature dimension of an approximated speech feature contour.

To generate the PCA bases of approximated speech feature contours, a training speech database is required so that eigenvectors of the approximated speech feature contours can be found. The goal of PCA is to search a linear combination of the original bases that maximizes the total variance of training approximated speech feature contours. By selecting the top *κ* principle bases or eigenvectors, PCA projection matrix is capable of representing approximated speech feature contour accurately with lower dimensions of projection coefficient vector.

To perform emotion identification, the PCA projection coefficients are utilized as the speech features. The PCA projection coefficients are computed by projecting the approximated speech feature contour onto the PCA bases. With the obtained PCA bases ${\overset{-}{v}}_{i}$, *i* = 1,2,…, *κ*, the PCA projection matrix is formed as $V=({\overset{-}{v}}_{1},{\overset{-}{v}}_{2},\ldots ,{\overset{-}{v}}_{\kappa })$. Let the approximated speech feature contour be denoted by $\overset{-}{a}={\left(a_{1},a_{2},\ldots ,a_{N}\right)}^{T}$, where *N* is the normalized contour length. Let ${\overset{-}{c}}_{\text{PCA}}$ refer to the PCA projection coefficient vector. The ${\overset{-}{c}}_{\text{PCA}}$ is computed by

(4) $$\begin{array}{c} {\overset{-}{c}}_{\text{PCA}}=V^{T}\overset{-}{a}\text{.} \end{array}$$

**Figure 6:**
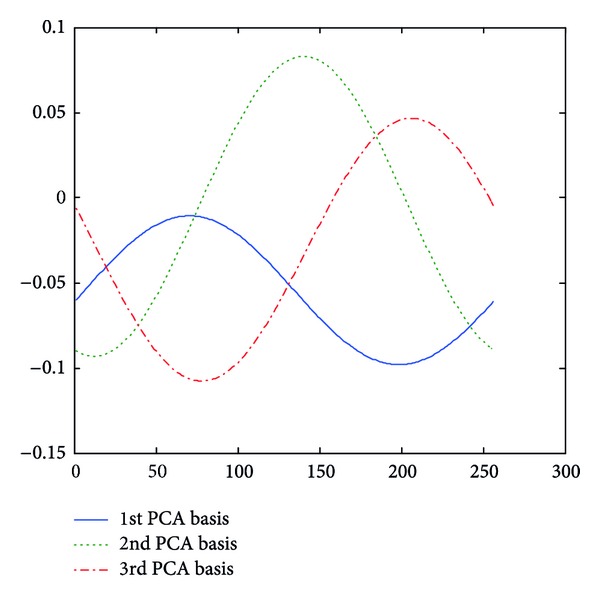
PCA bases of approximated LFE with *m* value being 1.

**Figure 7:**
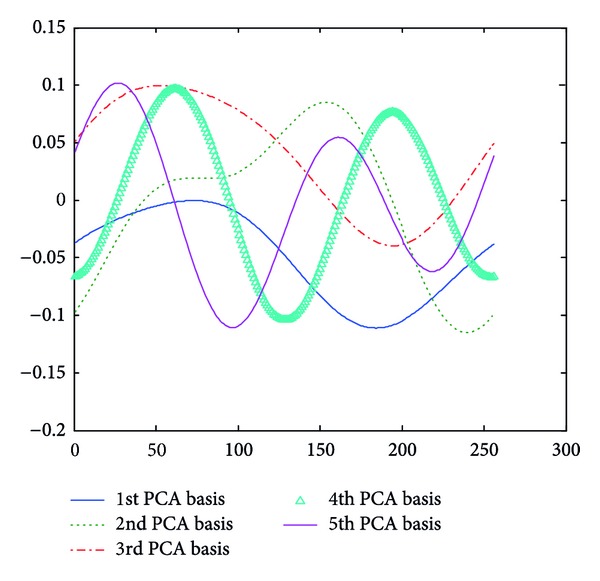
PCA bases of approximated LFE contours with *m* value being 2.

**Figure 8:**
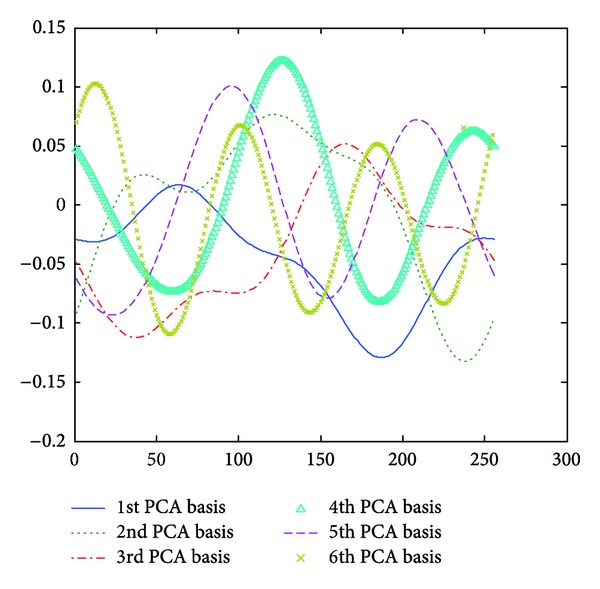
PCA bases of approximated LFE contours with *m* value being 3.

Take approximated LFE contour, for example; each *m* value (Section 3.2) associates with a set of PCA bases derived from the training approximated LFE contours. Figures 6, 7, and 8 give the PCA bases of approximated LFE contours with *m* value being 1, 2, and 3, respectively. It is noted that only significant PCA bases are shown in these figures.

## 4. Emotion Identification Using SVM

The SVM based on statistical machine learning is a powerful classifier [13, 19]. Using several crucial support vectors, the SVM has not only a clear structure but also a good classification performance. Considering data from two different classes, an SVM attempts to solve an optimization problem that finds a hyperplane that separates the data with maximum margin. Suppose the optimal separating hyperplane $(\overset{-}{w}\cdot \overset{-}{x})+b=0$, with $\overset{-}{w}\in R^{d}$ and *b* ∈ *R*, maximizes the margin $2/{||\overset{-}{w}||}^{2}$. A data point $\overset{-}{x}$ is then labeled *y* ∈ {1, −1} based on the decision function. To introduce kernel concepts, the separating hyperplane function in terms of the inner product of $\overset{-}{x}$ can be rewritten as

(5) f(x−)=sign⁡((w−·x−)+b)=sign⁡(∑i=1mαix−i·x−+b)=sign⁡(∑i=1mαik(x−,x−i)+b),

where *α* is a Lagrange multiplier, *i* is the number of vectors, and $k(\overset{-}{x},{\overset{-}{x}}_{i})$ is a kernel function. Using Mercer's theory, we can introduce a mapping function $\varphi \left(\overset{-}{x}\right)$, such that $k({\overset{-}{x}}_{j},{\overset{-}{x}}_{i})=\phi ({\overset{-}{x}}_{j})\phi ({\overset{-}{x}}_{i})$. This provides the ability of handling nonlinear data. Typical kernel functions include linear kernel, polynomial, and radial basis kernel.

The 2-class SVM is extended to multiclass emotion identification by one versus one technique. Totally, there are *C*_2_^*E*^ 2-class SVMs built, where *E* is the emotion class number.

## 5. Experimental Results

The database we used in this paper is German emotional speech database (GES) [20]. This database consists of seven emotion classes that are anger, joy, sadness, boredom, disgust, fear, and neutral and records utterances of five males and five females. Each speaker recorded ten speech utterances for each emotion. The number of speech files is about 800. Because we use only those speech files which are voted by over 80% voters, finally, the number of valid speech files is 535. The longest period of files is 8 seconds. The sampling rate of each file is 16 kHz with a resolution of 16 bits per sample. The frame size is 256 samples, with a 50% overlap in the two adjacent frames. There are 127 angry, 81 bored, 46 disgust, 69 fear, 71 joy, 79 neutral, and 62 sad files. Finally, 50% of the dataset was used for training and 50% for testing. We extracted features from both of the training and testing set.

First, performance of various approximated speech feature contours was evaluated. The experiment indicates approximated LFE contour has the best performance of them. Considering the approximated log energy contours, the *m* value of Section 3.2 is set as 1, 2, and 3. For each *m* value, only significant PCA bases are used. We choose 3, 5, and 6 significant PCA bases for *m* value equivalent to 1, 2, and 3, respectively. The emotion identification results using PCA projection coefficients are given in Table 1. We abbreviate the PCA projection coefficients as *P*_*m*_, which are *P*_*m*=1_, *P*_*m*=2_, and *P*_*m*=3_.

In Section 3.1, we introduce many statistical analysis features. In this paper, a feature selection procedure was conducted to choose an effective feature set for emotion identification. The chosen statistical analysis feature set after feature selection procedure is given in Table 2.

In the second experiment, the performance evaluation related to the adopted statistical analysis feature set as well as its combination with approximated LFE contours is summarized in Table 3. In this table, we abbreviate the adopted statistical analysis feature set as Γ. Moreover, the combination of the adopted statistical analysis feature set and approximated log energy contours are represented by Γ, *P*_*m*_. With the *P*_*m*_, the experimental result reveals that the identification rate of sadness is decreased slightly by 3.2%, but the identification rates of disgust, joy, and neutral are enhanced by 8.7%, 5.7%, and 5.2%, respectively. The Γ, *P*_3_ feature set achieves the best total identification rate 82.3%, which is 2.26% higher than merely using statistical analysis feature set Γ. The confusion matrix corresponding to the optimal feature set Γ, *P*_3_ is given in Table 4.

## 6. Conclusion

This work proposes a method to generate approximated speech feature contours using forward and inverse Fourier transform. PCA projection coefficients provide an efficient feature representation of the approximated speech feature contours. For PCA projection coefficients of approximated log frame energy contour, 44.39% average identification rate can be achieved. After integrating PCA projection coefficients with the selected statistical analysis feature set, the average identification rate coefficients are increased from 80% to 82.26%. This result demonstrates the effectiveness of the proposed PCA projection coefficients generated from approximated speech feature contours. In the future, the effectiveness of other different Fourier coefficients can be exploited. Moreover, wrapper selection and linear discriminant analysis may further increase the performance.

## Figures and Tables

**Table 1:** Performance evaluation using approximated LFE contours.

Feature	Dimension	Identification rate (%)
*P*_*m*=1_	3	44.9
*P*_*m*= 2_	5	43.77
*P*_*m*=3_	6	44.52

**Table 2:** Adopted statistical analysis features after feature selection.

Statistical analysis feature set	Dim.
Silence ratio	1
Voiced ratio	1
Mean and standard deviation of pitch	2
Mean and standard deviation of log frame energy	2
Mean and standard deviation of subband powers	8
Mean and standard deviation of spectral centroid	2
Mean and standard deviation of bandwidth	2
Mean and standard deviation of MFCCs	26

**Table 3:** Performance evaluation using different feature sets.

	Ang.	Bor.	Dis.	Fear	Hap.	Neu.	Sad.	Total
Γ	93.7	80	69.6	76.5	62.9	76.9	87.1	80.0
Γ, *P*_1_	93.7	80	69.6	76.5	65.7	79.5	87.1	80.8
Γ, *P*_2_	93.7	80	73.9	79.4	65.7	82.1	83.9	81.5
Γ, *P*_3_	93.7	80	78.3	79.4	68.6	82.1	83.9	82.3

**Table 4:** Confusion matrix of Γ, *P*_3_ feature set. The left column denotes actual emotions, and the top row represents predicted emotions.

	Ang.	Bor.	Dis.	Fear	Hap.	Neu.	Sad.
Ang.	59	0	0	1	3	0	0
Bor.	0	32	0	0	0	7	1
Dis.	2	0	18	1	1	0	1
Fear	4	0	0	27	1	2	0
Hap.	8	0	0	3	24	0	0
Neu.	0	7	0	0	0	32	0
Sad.	0	2	0	0	1	2	26
